# Oral and Skin Sensitisation to Peanut Show Different Immunological Features in Brown Norway Rats

**DOI:** 10.1111/sji.70069

**Published:** 2025-11-17

**Authors:** Tiffany K. S. Sztuk, Jeppe M. Larsen, Neil M. Rigby, Anne‐Sofie R. Ballegaard, Irina Pozdnyakova, Stef J. Koppelman, Alan R. Mackie, Katrine L. Bøgh

**Affiliations:** ^1^ National Food Institute Technical University of Denmark Kgs. Lyngby Denmark; ^2^ School of Food Science & Nutrition University of Leeds Leeds UK; ^3^ NNF Center for Protein Research University of Copenhagen Copenhagen Denmark; ^4^ Institute of Agriculture and Natural Resources, Food Science and Technology University of Nebraska‐Lincoln Lincoln Nebraska USA

**Keywords:** Ara h, IgE‐binding epitopes, immune cell profiling, oral sensitisation, skin sensitisation

## Abstract

Food allergy may develop after both oral and skin exposure to food allergens. Identifying immunological features associated with the sensitisation route is important for understanding the extent to which patients are sensitised through the oral or skin route and may be required to improve food allergy immunotherapy. Thus, the present study aimed at comparing the immunological features of peanut sensitisation induced through the oral and skin route in Brown Norway (BN) rat models. Sensitisation to peanut protein extract (PPE) was induced via oral or skin administration in BN rats. Allergy development was analysed by ELISA for PPE‐ and major allergen‐specific IgE levels and by ear swelling responses to native and denatured PPE. Intestinal and skin tissues were analysed by flow cytometry for immune cell compositions and by TaqMan PCR for the expression of cytokines. Oral and skin sensitisation showed distinct patterns of specific IgE against the major peanut allergens. Conformational IgE epitopes dominated both routes of sensitisation. Skin sensitisation was associated with the activation of T cells and the expansion of antigen presenting cells in both the intestine and skin, whereas oral sensitisation showed a limited effect on immune cell composition and activation. Oral and skin sensitisation were associated with different intestinal cytokine expression profiles. Oral and skin sensitisation drive different responses to the major peanut allergens and promote different immunological responses in the intestine.

## Graphical abstract

Immunological features differ between oral and skin sensitisation, with oral and skin sensitisation driving the development of IgE to different major allergens in peanut, which could pave the way for determining the sensitisation route in patients. Further, oral and skin sensitisation promote different immunological responses in the intestine, which could influence disease expression. Graphical abstract was created with BioRender.com.

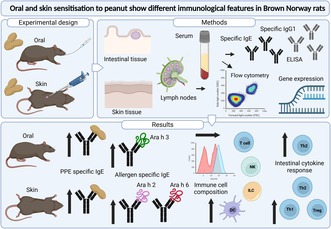

## 1 Introduction

Food allergy is driven by an abnormal immune response to proteins in foods [1]. The severity of allergic reactions varies depending on the causal food and may lead to fatal systemic anaphylaxis [2]. Peanut allergy (PA), which is exclusively mediated by peanut‐specific IgE [3], is the most prevalent cause of anaphylaxis compared to any other food [4], highlighting the importance of understanding the mechanisms behind peanut sensitisation. The understanding of peanut sensitisation is largely limited to knowledge derived from animal studies, as humans cannot be experimentally sensitised for ethical reasons [5, 6]. Traditionally, ingestion was believed to be the main cause of sensitisation to peanuts, as the gastrointestinal (GI) tract is the primary route of exposure to food allergens [7]. However, data from cohort studies suggest that sensitisation to peanuts can develop through the skin, as environmental peanut exposure has been linked to PA with increased risk in patients with atopic dermatitis [8, 9, 10]. Interestingly, mechanistic studies in humans indicate that allergic sensitisation is the default immune response of skin, further supporting a role of this sensitisation route in PA [11]. Animal studies further support sensitisation through the skin route, as epicutaneous exposure of peanut on intact or damaged skin readily induces peanut‐specific IgE [12, 13]. Despite these findings pointing to the skin as an important sensitisation route, many animal studies have likewise shown that sensitisation can be induced via the oral route albeit requiring the use of adjuvant [14, 15]. Collectively, experimental data suggest that sensitisation to food can occur both orally and via the skin. It seems reasonable to assume that the nature of the immune response to food allergens is influenced by sensitisation route, especially when taken into consideration that the GI tract promotes digestion of food proteins into peptide fragments, whereas the skin does not possess this type of digestive machinery. However, it remains unknown if sensitisation via oral and skin routes leads to different immunological features, for example, in terms of antibody responses to allergens, epitope recognition and cellular mechanisms. Elucidating such features may be important in identifying the route of sensitisation in humans after disease development for targeting potential different underlying mechanisms via novel personalised treatment strategies [13, 16]. Here, we aimed to investigate the immunological features associated with sensitisation to peanuts via the oral and skin routes in Brown Norway (BN) rats.

## 2 Materials and Methods

### 2.1 Peanut Products

Peanut protein extract (PPE) and purified major allergens Ara h 1, Ara h 2, Ara h 3 and Ara h 6 were prepared and characterised as previously described [16]. The relative amount of the major allergens in PPE was approx. 10% Ara h 1, 10% Ara h 2, 45% Ara h 3 and 10% Ara h 6 as determined by size exclusion chromatography. PPE and purified peanut allergens, as well as their digoxigenin (DIG)‐coupled versions, were denatured by reduction and alkylation, and protein unfolding was confirmed by circular dichroism (CD) as previously described [17].

### 2.2 Animals

BN rats were from the in‐house breeding colony at the National Food Institute, Technical University of Denmark (DTU), Denmark and housed as previously described [18, 19]. Animals were kept on an in‐house produced diet free from legumes for ≥ 14 generations to avoid tolerance to proteins homologous to peanut proteins, as previously described [16]. Animal experiments were approved by the Danish Animal Experiments Inspectorate (authorisation numbers 2015‐15‐0201‐00553‐C1 and 2020‐15‐0201‐00500‐C1) and overseen by the DTU Animal Welfare Committee for animal care and use.

### 2.3 Sensitisation Regimens

#### 2.3.1 Oral

**FIGURE 1:**
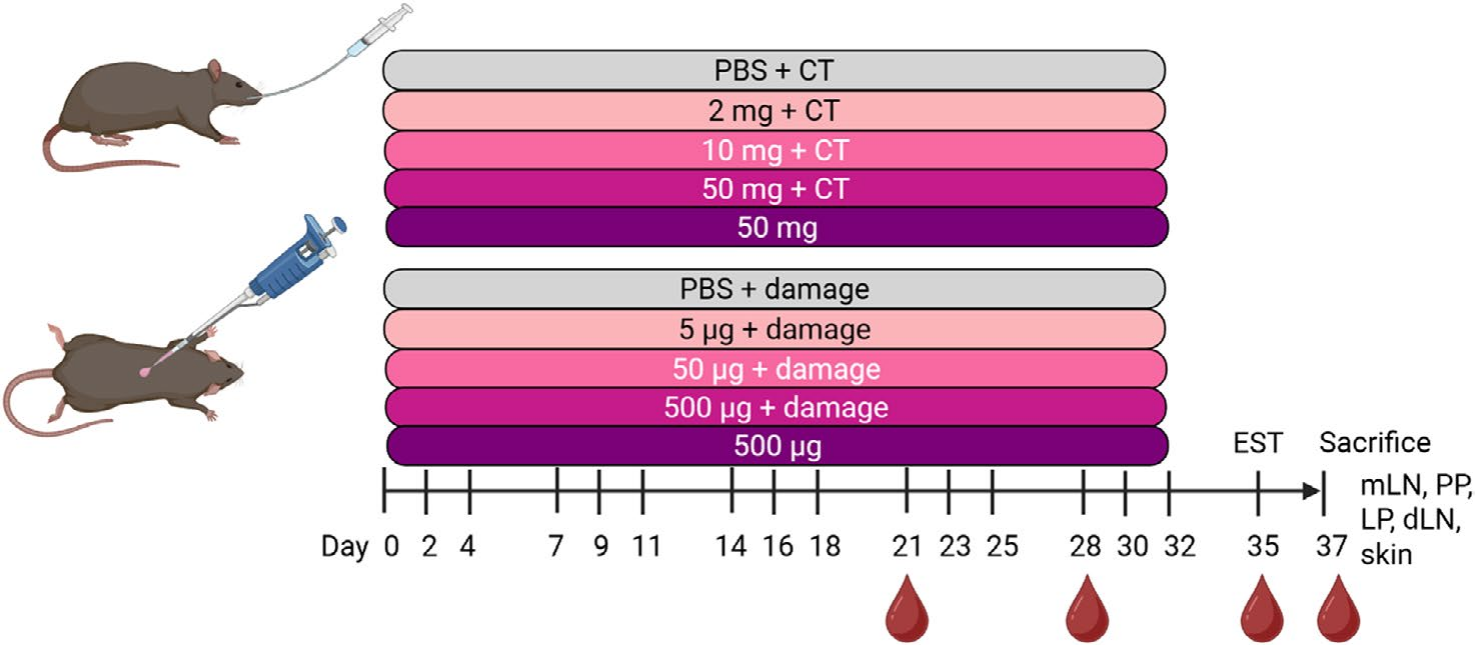
Design of animal experiments for sensitisation through the oral and skin route. Brown Norway rats (*n* = 8/group) were dosed with either PBS as control or with three different doses of peanut protein extract (PPE) via either oral gavage or skin application. Cholera toxin (CT) was co‐administered with PPE through oral route, whereas mechanical disruption of the skin was induced prior to skin application for the skin route, in the indicated groups. All doses were administered three times per week (Mondays, Wednesdays and Fridays) from Day 0 to Day 32. On Day 35, an ear swelling test (EST) was performed for control groups and groups of animals sensitised to PPE. On Day 37, animals were sacrificed and small intestine (SI), lamine propria (LP). Peyer's patches (PP), mesenteric lymph nodes (mLN), skin draining lymph nodes (dLN) and skin samples were collected. Blood samples were collected throughout the study as indicated. Figure created with BioRender.com.

Female BN rats, 4–6 weeks of age (*n* = 8/group) were exposed to either 0 mg (control), 2 mg, 10 mg or 50 mg of PPE in 0.5 mL PBS (137 mM NaCl, 3 mM KCl, 8 mM Na_2_HPO_4_, 1 mM KH_2_PO_4_, pH 7.2) with 20 μg cholera toxin (CT; 100B, List Biological Laboratories Inc., Campbell, CA, USA) or to 50 mg of PPE in 0.5 mL PBS without CT by oral gavage. Animals were dosed three times per week (Mondays, Wednesdays and Fridays) for five consecutive weeks (Figure 1).

#### 2.3.2 Skin

Female BN rats, 4–8 weeks of age (*n* = 8/group) were exposed to either 0 μg (control), 5 μg, 50 μg or 500 μg of PPE in PBS on damaged skin or to 500 μg of PPE in PBS on intact skin. Animals were dosed three times per week (Mondays, Wednesdays and Fridays) for five consecutive weeks (Figure 1). Damaged skin was induced once every week by removing the hair on the abdomen with an electric shaver (Oster, PowerPro Ultra, blade 50) followed by scratching the skin using sandpaper (grain size P180) as previously described [20]. Subsequently, 100 μL of the specified PPE doses were applied on a 2 × 2 cm^2^ area on the shaved abdominal skin. To avoid oral exposure, the skin application area was covered with an elastic gauze bandage wrapped around the abdomen, and animals were placed alone in a cage for 1 h. Subsequently, the skin application area was rinsed with water and animals were placed in their original cages. Induction of skin damage was confirmed by measurements of increased transepidermal water loss (TEWL) (see Figure S1).

### 2.4 Ear Swelling Test, Oral Challenge and Sampling

Blood samples were collected on Days 21, 28, 35 and 37 (Figure 1). Blood samples were converted to sera and stored at −20°C until analysis. Animals were subjected to an ear swelling test (EST) on Day 35 with 10 μg of PPE or denatured PPE (d.PPE) in 20 μL PBS in the right and left ear, respectively, performed as previously described [18]. On Day 37, BN rats were subjected to an oral challenge with 100 mg PPE in 1 mL PBS by oral gavage, and animals were euthanised 30 min after the challenge by exsanguination using carbon dioxide inhalation as anaesthesia. Whole blood, small intestinal (SI), Peyer's patches (PP), mesenteric lymph nodes (mLN), skin draining lymph nodes (dLN) and skin samples were collected for analysis.

### 2.5 ELISA—Quantification of Antibody Titres

IgG1 and IgE specific for native or denatured versions of PPE and purified major peanut allergens were quantified by means of indirect and antibody‐capture ELISAs, respectively, as previously described [16] (Appendix S1).

### 2.6 Flow Cytometry

Single‐cell suspensions were prepared from SI, PP, mLN, dLN, skin and whole blood samples and analysed by flow cytometry, as previously described [21, 22] and further detailed in Appendix S1. Three different staining panels were used to analyse antigen presenting cells (APCs), T regulatory cells (Tregs) and T cell skin and intestine homing receptor expression. Please refer to Figures S5–S7 for gating strategies and definition of cell populations.

### 2.7 Intestinal and Skin Gene Expression

The mRNA gene expression levels of selected cytokines (TSLP, IL‐25, IL‐33, IL‐4, IL‐5, IL‐13, IL‐10, IFN‐γ and TGF‐β) and the tight junction protein occludin (Ocln) were analysed in SI and skin samples by RT‐qPCR using the TaqMan protocol, as described previously [21] and detailed in Appendix S1.

### 2.8 Statistical Analyses

Graphs and statistical analyses were generated using Prism version 9.3.1 (GraphPad, San Diego, CA, US). Normal distribution of data was assessed by D'Agostino–Pearson normality test. Kruskal–Wallis test followed by Dunn's post‐test was performed for analysing multiple comparisons between medians from more than two groups. Statistical comparisons between two groups were analysed by Mann–Whitney test or Student's *t* test. Differences between paired data were compared by using the Wilcoxon matched‐pairs signed rank test. Data from EST was analysed by Welch's test. Multiple *t*‐testing was applied for analysing differences between sensitised and control animals in different compartments in the flow cytometric analyses. Asterisk(s) indicate statistically significant difference between two given groups: *p ≤ 0.05, **p ≤ 0.01 and ***p ≤ 0.001, ****p ≤ 0.0001.

## 3 Results

### 3.1 Sensitisation via Oral and Skin Routes Is Dose‐Dependent

**FIGURE 2:**
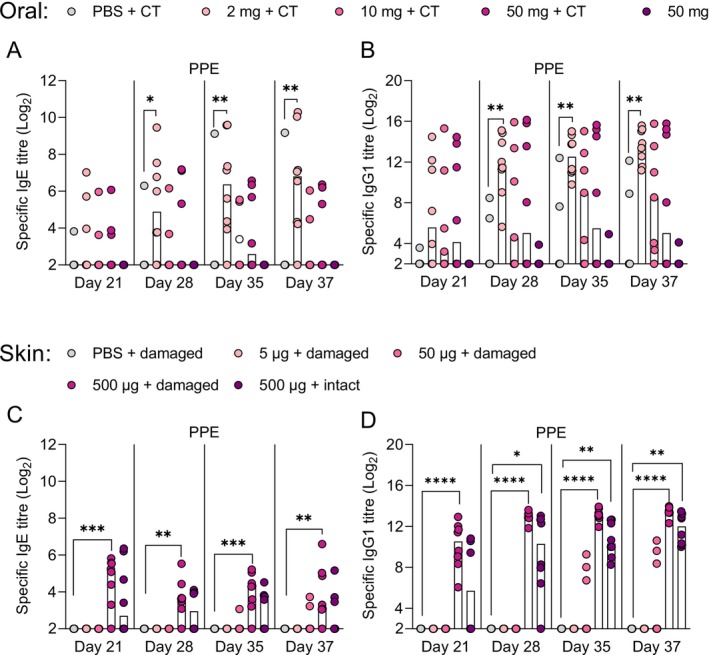
Peanut protein extract (PPE)‐specific IgE and IgG1 levels following PPE administration via the oral and skin route. A dose–response study with PPE was conducted with oral (top panel) or skin administration (bottom panel), where cholera toxin (CT) or mechanical disruption of the skin (damaged skin) was employed for oral and skin administration, respectively, in indicated groups. The levels of PPE‐specific IgE (A, C) and IgG1 (B, D) were assessed by ELISA on Days 21, 28, 35 and 37. Each symbol represents a single animal, and horizontal lines indicate the median of each group. Statistically significant differences compared to control animals receiving PBS are indicated with asterisk(s), *p < 0.05, **p < 0.01, ***p < 0.001, ****p < 0.0001.

It remains largely unknown, if sensitisation to food allergens via the oral and skin route is associated with different underlying immunological features. Thus, here we conducted two dose–response studies using PPE administration via either oral or skin exposure in BN rats to compare route‐specific peanut sensitisation patterns (Figure 1). Peanut sensitisation was dose‐dependent for both oral and skin sensitisation (Figure 2A,C), with oral administration of 2 mg PPE together with CT or application of 500 μg PPE on damaged skin inducing statistically significant levels of PPE‐specific IgE when compared to control animals. The pattern of the PPE‐specific IgG1 levels was similar to the PPE‐specific IgE levels (Figure 2B,C). Further, the specific IgG1 and IgE responses against the individual major allergens Ara h 1, Ara h 2, Ara h 3 and Ara h 6 showed an overall dose–response pattern similar to that of PPE‐specific IgG1 and IgE (Figures S2 and S3). The two sensitised groups (orally gavaged with 2 mg PPE together with CT and application of 500 μg PPE on damaged skin) were included in the following analyses for further assessment of the immunological features associated with oral and skin sensitisation.

### 3.2 The Primary Peanut Allergens Giving Rise to IgE Differ Between Oral and Skin Sensitisation

**FIGURE 3:**
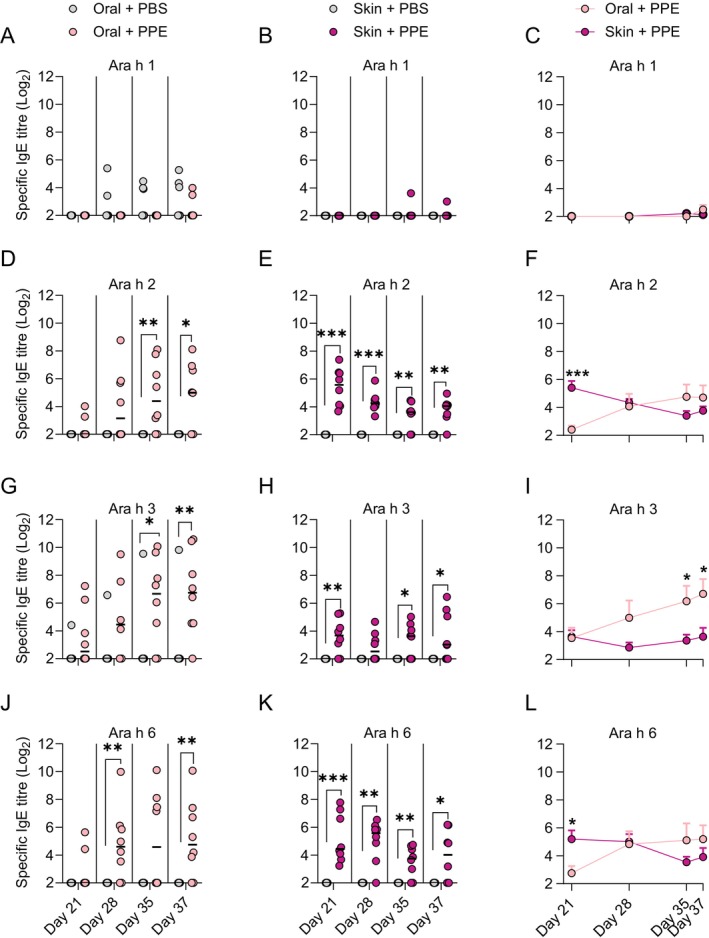
Major allergen‐specific IgE levels during sensitisation via the oral and skin route. The levels of Ara h 1‐, Ara h 2‐, Ara h 3‐ and Ara h 6‐specific IgE in orally (A, D, G J) and skin sensitised (B, E, H K) animals were determined by ELISA on Days 21, 28, 35 and 37. Left and middle panel: Each symbol represents a single animal, and horizontal lines indicate the median of each group. Mean levels of Ara h 1‐, Ara h 2‐, Ara h 3‐ and Ara h 6‐specific IgE in orally and skin sensitised animals were compared (C, F, I L). Right panel: Each symbol and bar represent the mean and SEM, respectively, of each group at a given time point. Statistically significant differences compared to control animals receiving PBS are indicated with asterisk(s), *p < 0.05, **p < 0.01, ***p < 0.001.

The oral and skin route of sensitisation may be associated with different sensitisation patterns to the major allergens in peanut. Both oral and skin sensitisation were associated with sensitisation to Ara h 2, Ara h 3 and Ara h 6, but not Ara h 1 (Figure 3A,B,D,E,G,H,J,K). However, the pattern of the allergen‐specific IgE levels differed between the two routes, with Ara h 3 being the dominating allergen in animals orally sensitised (Figure 3I), and Ara h 2 and Ara h 6 being the dominating allergens with early induction in animals sensitised through the skin (Figure 3F,L). Furthermore, the course of sensitisation differed between the two routes of sensitisation. Ara h 2, Ara h 3 and Ara h 6‐specific IgE levels increased over the course of the study for orally sensitised animals, whereas the Ara h 2 and Ara h 6‐specific IgE levels already peaked at Day 21 and then decreased for skin sensitised animals. These findings indicate a distinct allergen‐specific IgE pattern and progression following sensitisation via the oral and skin route, which may arise due to differences in the interaction between the major allergens and the tissue‐specific immune system.

### 3.3 Conformational IgE Epitopes Dominate Both Oral and Skin Sensitisation

**FIGURE 4:**
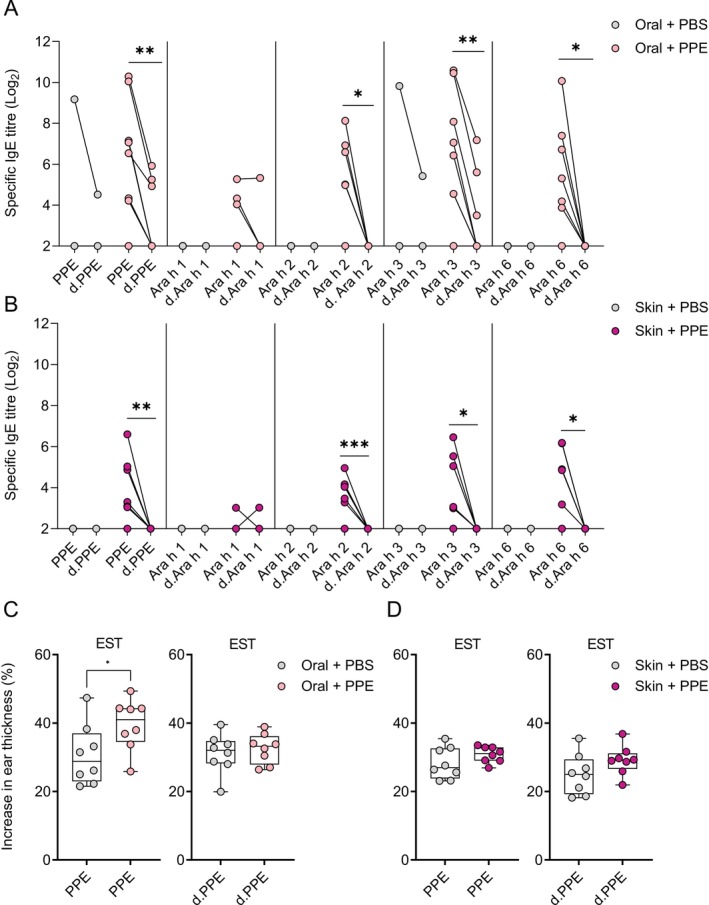
The epitope recognition profile of IgE in animals sensitised to peanut protein extract (PPE) via the oral or skin route. The levels of IgE directed against native (containing both conformational and linear epitopes) allergens (PPE, Ara h 1, Ara h 2, Ara h 3 and Ara h 6) and denatured (d.) containing only linear epitopes (conformational epitopes removed) allergens (d.PPE, d.Ara h 1, d.Ara h 2, d.Ara h 3 and d.Ara h 6) in rat sera determined by ELISA on Day 37 of animals sensitised orally (A) or via the skin (B). On Day 35, sensitised animals were subjected to an ear swelling test (EST) with PPE or d.PPE (C, D). Statistically significant differences are indicated with asterisk(s), *p < 0.05, **p < 0.01, ***p < 0.001.

The preferential development of allergen‐specific IgE towards linear or conformational epitopes could be dependent on the sensitisation route, for example, due to digestion and denaturation of proteins likely occurring to a larger degree in the GI tract compared to the skin. Here, IgE and IgG1 were measured against native (containing both conformational and linear epitopes) and denatured (containing only linear epitopes (conformational epitopes removed)) versions of PPE and the major allergens to evaluate the degree of recognition of conformational versus linear epitopes for orally and skin sensitised animals. Ara h 2‐ and Ara h 6‐specific IgE were raised exclusively against conformational epitopes after both oral and skin sensitisation. In contrast, whereas Ara h 3‐specific IgE was raised exclusively against conformational epitopes after skin sensitisation, both linear and conformational epitopes were detected after oral sensitisation, though the recognition of conformational epitopes was more pronounced (Figure 4A,B). IgG1 responses revealed a similar pattern of conformational versus linear epitopes for PPE and the major peanut allergens (Figure S4), with conformational epitopes being the primary inducer of IgG1 after both oral and skin sensitisation. However, in contrast to the IgE response, Ara h 2‐ and Ara h 6‐specific IgG1 were not exclusively raised against conformational epitopes, indicating a broader IgG1 epitope pattern, but possibly with a preferential role for conformational epitopes in peanut allergy no matter the route of sensitisation. The ear swelling test (EST) response to native PPE was higher in animals sensitised via the oral, but not skin, route compared to control animals (Figure 4C). In contrast, no EST response was observed to denatured PPE (Figure 4D), further highlighting a central role for conformational epitopes in the clinical response to PPE. Collectively, irrespective of the sensitisation route to peanut, the recognition of conformational epitopes was greater than the recognition of linear epitopes, thus underpinning a central role of conformational epitopes in peanut sensitisation.

### 3.4 Skin, but Not Oral, Sensitisation Affects Immune Cell Composition in Skin and Intestine

**FIGURE 5:**
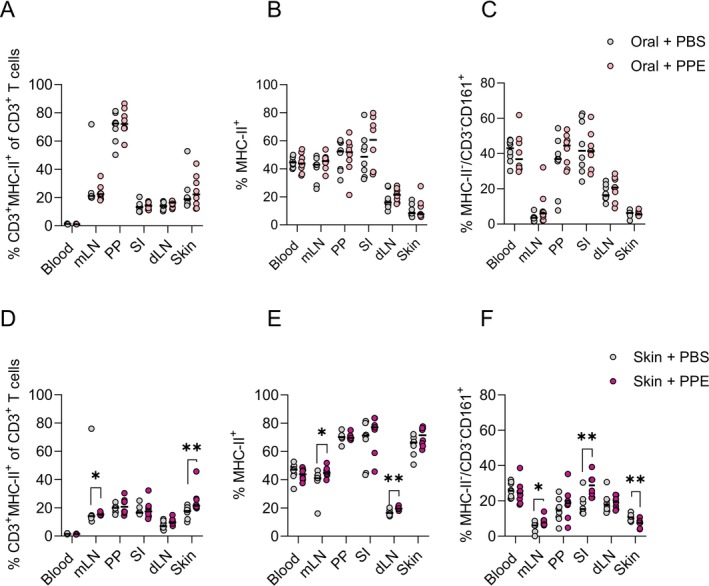
Flow cytometric analyses for the identification of immune cell populations induced in response to oral and skin sensitisation. The percentage of MHC‐II activated T cells out of the total T cell population in blood, mesenteric lymph nodes (mLN), Peyer's patches (PP), small intestine (SI), skin draining lymph nodes (dLN) and skin of orally (A) and skin (D) sensitised animals. The percentage of antigen presenting cells in blood, mLN, PP, SI, dLN and skin of orally (B) and skin (E) sensitised animals. The percentage of immune cells expressing the CD161 marker in blood, mLN, PP, SI, dLN and skin of orally (C) and skin (F) sensitised animals. Each symbol represents a single animal, and horizontal lines indicate median of each group. Statistically significant differences compared to control animals receiving PBS are indicated with asterisk(s), *p < 0.05, **p < 0.01.

Previous studies indicate an immunological skin‐to‐gut crosstalk, which may play a role in the expression of food allergy [23, 24, 25, 26]. Here, we assessed the frequency of various immune cell populations in blood, mLN, PP, SI, skin dLN and skin samples in orally and skin sensitised animals in comparison to control animals. Flow cytometric analyses were performed using three staining panels focusing on the frequencies of APCs, T cell phenotypes (Treg and Th1), and T cell expression of skin and intestine homing markers. Overall, oral sensitisation showed a limited effect on the composition of immune cells in the analysed tissues (Figure 5A–C and Figures S8–S10). On the contrary, skin sensitisation influenced the composition of immune cells in both the skin and intestine. Skin sensitisation was associated with the activation of T cells (MHC‐II expression) in mLN and skin (Figure 5D), as well as the expansion of MHC‐II^+^ APCs in the mLN and dLN (Figure 5E). Furthermore, skin sensitisation was associated with higher frequencies of MHC‐II^−^CD3^−^CD161^+^ cells in the mLN and SI, and lower frequencies in the skin, indicating the expansion of a natural killer or innate lymphoid cell‐like population in the intestine (Figure 5F). No differences were observed in the frequencies of Tregs or T cells expressing skin (CCR4) or intestinal (CCR9) homing markers (Figures S9 and S10). Collectively, our findings demonstrate that skin sensitisation affects immune cell composition in both the skin and intestine to a much larger extent than oral sensitisation.

### 3.5 Oral and Skin Sensitisation Drive Different Cytokine Profiles in the Intestine

**FIGURE 6:**
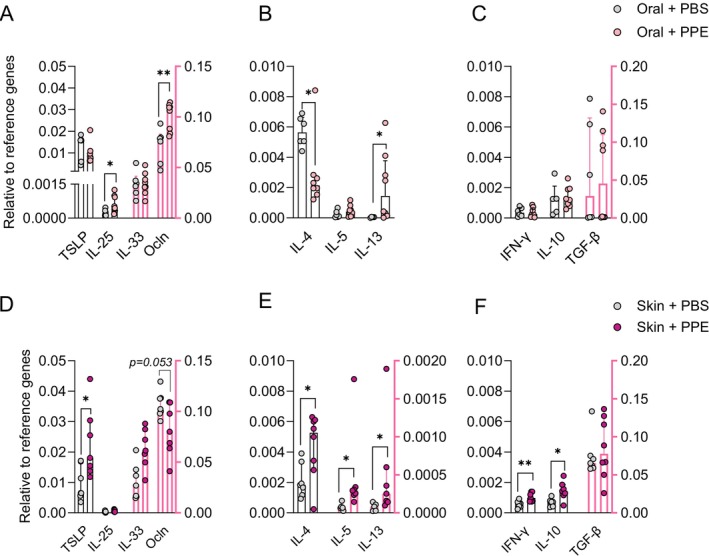
Small intestinal gene expression in animals sensitised to peanut protein extract through the oral (top panel) or skin route (bottom panel). Relative gene expression of alarmins (thymic stromal lymphopoietin (TSLP), interleukin (IL)‐25 and IL‐33) and expression of a tight junction protein (Occludin (Ocln)) (A, D). Relative gene expression of T helper cell 2 cytokines (IL‐4, IL‐5 and IL‐13) (B, E). Relative gene expression of T helper cell 1 (interferon‐gamma (IFN‐γ)) and regulatory T cell cytokines (IL‐10 and transforming growth factor (TGF)‐β) (C, F). Gene expression levels are determined relatively to two reference genes. Each symbol represents a single animal, and bars with upper interquartile range indicate median of each group. Median bars in black are plotted on the left *y*‐axis and median bars in pink are plotted on the right axis. Statistically significant differences compared to control animals receiving PBS are indicated with asterisk(s), *p < 0.05, **p < 0.01.

We assessed the cytokine profiles in the small intestine (SI) and skin to further investigate whether the observed differences in the immune cell profiles of orally and skin sensitised animals were associated with functional differences. mRNA gene expression of type‐2 alarmins (TSLP, IL‐25 and IL‐33), type‐2 effector cytokines (IL‐4, IL‐5 and IL‐13), the type‐1 effector cytokine IFN‐γ, regulatory cytokines (IL‐10 and TGF‐β), and the tight junction molecule occludin (Ocln) were analysed in SI (Figure 6) and skin (Figure S11) tissue samples from animals sensitised via the oral and skin route. Animals orally sensitised showed a statistically significant higher Ocln and IL‐25 expression in SI tissue compared to control animals (Figure 6A). Contrary, skin sensitised animals showed a markedly lower expression of Ocln and a statistically significant higher expression of TSLP compared to control animals (Figure 6D). IL‐4, IL‐5 and IL‐13 expression was statistically significant higher in SI tissue of animals sensitised via the skin, whereas only IL‐13 was statistically significant higher and IL‐4 lower in animals orally sensitised when compared to control animals (Figure 5B,E). No statistically significant differences were observed for the expression of type‐1 or regulatory cytokines between orally sensitised and control animals (Figure 5C). In contrast, statistically significant higher levels of IFN‐γ and IL‐10 compared to control animals were demonstrated for animals sensitised via the skin (Figure 5F). Collectively, these results indicate that oral sensitisation mainly induces cytokines associated with a type‐2 immune response, whereas skin sensitisation induces cytokines derived from a broader spectrum of type‐2, type‐1 and regulatory immune responses. The route of sensitisation showed only limited effects on cytokine expression in skin samples (Figure S11). These results show that both oral and skin sensitisation mainly affect the intestinal cytokine milieu.

## 4 Discussion

Sensitisation to food proteins was originally considered to occur mainly through the GI tract; however during the last decade sensitisation via the skin has received considerable attention [12, 23, 27]. Dissecting the underlying mechanisms of route‐dependent sensitisation may allow the identification of biomarkers for deciphering the route by which patients have been sensitised, further enabling targeted development of effective preventive and treatment strategies for food allergy. Thus, the present study aimed at investigating the underlying immunological features of sensitisation induced by the oral and skin route in BN rat models of PA.

BN rat models of oral and skin sensitisation were established with conditions giving rise to the induction of PPE‐specific IgE. Interestingly, Ara h 3 was the dominating allergen for driving sensitisation through the oral route, whereas Ara h 2 and Ara h 6 were the dominating allergens for driving sensitisation through the skin. This finding indicates that peanut sensitisation is driven by distinct major allergens depending on the route of exposure and therefore leads to distinct allergen‐specific IgE patterns. Indeed, it has been shown that peanut allergic patients have a heterogeneous allergen‐specific IgE binding pattern [28, 29, 30], which may then be related to differences in the route of sensitisation. The distinct pattern of allergen‐specific IgE responses in orally and skin sensitised animals was clearly not attributable to the amount of individual allergens in our PPE preparation, as Ara h 3 was the most abundant allergen followed by equal amounts of Ara h 1, Ara h 2 and Ara h 6. Instead, other contributing factors may be decisive for the observed differences, including the physicochemical properties of the individual allergens. Digestibility is considered to influence food protein allergenicity [31, 32], and a higher level of digestion of allergen via the oral route could explain differences in the sensitisation pattern. However, digestibility cannot explain why we observe higher Ara h 3‐specific IgE levels via the oral route compared to the skin route, since Ara h 3 in general is considered more susceptible to digestion than Ara h 2 and Ara h 6 [33]. Thus, other physicochemical properties of the allergens may explain the route‐specific IgE patterns of sensitisation.

The preferential development of IgE against conformational or linear epitopes could be affected by the sensitisation route, since allergen unfolding and digestion likely occur via the oral route leading to linear fragments. Here, we assessed the contribution of conformational versus linear epitope‐binding in orally and skin sensitised animals. Conformational IgE‐binding epitopes dominated in both orally and skin sensitised animals regardless of allergen, indicating that the sensitisation route did not affect the preference for conformational or linear IgE‐binding epitopes. This was unexpected, since patients with peanut allergy show a diverse and patient‐specific IgE recognition pattern of Ara h 2 and Ara h 6 conformational as well as linear epitopes [34, 35, 36] that could be linked to the sensitisation route. Interestingly, linear Ara h 2 and Ara h 6 epitope patterns have previously been linked to peanut allergy severity [37]. Thus, further dissecting both conformational and linear epitope recognition patterns of the major peanut allergens may in the future contribute to revealing route‐specific sensitisation.

Sensitisation to food allergens is a highly complex immunologic reaction involving many different immune cells and molecules. Here, we investigated if sensitisation via the oral or skin route affected the composition of various immune cells and expression of cytokines in the intestine and skin. Skin sensitisation affected immune cell composition in both the skin and intestine, whereas oral sensitisation had little effect on immune populations in these tissues. Further, both oral and skin sensitisation had little effect on the expression of cytokines in the skin. However, oral sensitisation was associated with increased expression of type‐2 cytokines, whereas skin sensitisation was associated with increased expression of type‐2, type‐1 and regulatory cytokines, as well as lower expression of occludin. Although these findings may be difficult to translate into biomarkers for sensitisation route, they highlight that different immunological mechanisms are in play for sensitisation via the oral and skin routes. These mechanisms may have the potential to affect the outcome of sensitisation, such as the sensitisation pattern to the major peanut allergens, as reported here. Our findings are in line with previous research demonstrating the existence of a skin‐gut axis that can promote food allergy by expansion of intestinal mast cells in response to mechanical skin injury [38]. Interestingly, here we demonstrated activation of T cells and expansion of APCs, which indicate that skin sensitisation also affects adaptive immune responses in the intestine. The intestinal immune profile may also influence the expression of food allergy upon subsequent oral exposure to the allergen; thus sensitisation route may influence clinical reactivity. Indeed, it has been demonstrated that skin sensitised mice experienced anaphylaxis upon a subsequent single oral dose, whereas orally sensitised mice did not [13]. This subsidises that a skin‐derived sensitisation profile may promote severe allergic phenotypes. Interestingly, we found that Ara h 2 and Ara h 6 were the dominating allergens in skin sensitised animals, and these are indeed considered the most clinically relevant allergens, and thus, possibly markers of disease severity [39, 40, 41]. We also found that IL‐4, IL‐5 and IL‐13 were increased in the intestine of skin sensitised animals, whereas only IL‐13 was increased in orally sensitised animals. These findings support that skin sensitisation profile may drive a more severe allergic phenotype due to several types of effector cytokines in the intestine.

Targeting the underlying immune mechanisms of route‐specific sensitisation to peanut may be necessary for the optimal treatment of PA. Allergen‐specific immunotherapy (IT) is the only treatment that can modify the allergen‐specific IgE response and potentially cure food allergies [42]. It has been shown that IT via the oral, epicutaneous and sublingual routes induces desensitisation in orally peanut sensitised mice, but the persistency of desensitisation varies between the routes possibly due to the induction of different Tregs subsets [43]. Different routes of IT may thus be required to modify allergic responses induced by oral or skin sensitisation.

It is important to notice that the current study is descriptive in nature and additional studies are needed to ascertain causal mechanisms. Furthermore, we used a BN rat model that may not fully capture the complex human immune responses to food allergens. Thus, further detailed and comparative studies are warranted. To conclude, our results demonstrate that the oral and skin sensitisation routes drive different immunological features related to both humoral and cellular immune responses leading to PA development.

## Author Contributions

Conceptualisation: K.L.B., J.M.L. and T.K.S.S. Funding acquisition: K.L.B. Investigation and data curation: T.K.S.S., J.M.L. and K.L.B. Methodology: K.L.B., J.M.L., A.‐S.R.B. and T.K.S.S. Visualisation: T.K.S.S., J.M.L. and K.L.B. Formal analysis: T.K.S.S. Resources: I.P., N.M.R., S.J.K. and A.R.M. Supervision: K.L.B. and J.M.L. Writing – original draft: T.K.S.S. and J.M.L. Writing – review and editing: K.L.B. All authors made substantial intellectual contributions to the study, reviewed the manuscript critically and approved the final version of the manuscript.

## Conflicts of Interest

T.K.S.S. is currently an employee at ALK. K.L.B. has an ongoing collaboration with ALK. The other authors declare that the research was conducted in the absence of any commercial or financial relationships that could be construed as a potential conflicts of interest.

## Supporting information


**Appendix S1:** sji70069‐sup‐0001‐AppendixS1.docx.

## Data Availability

The data that support the findings of this study are available from the corresponding author upon reasonable request.
