# Supplementary material for: Oral and Skin Sensitisation to Peanut Show Different Immunological Features in Brown Norway Rats
Source: Scand J Immunol. 2025 Nov 17;102(5):e70069. doi: 10.1111/sji.70069 (PMC12623298; doi:10.1111/sji.70069)
Supplement: Supplementary file 1 — Appendix S1: sji70069‐sup‐0001‐AppendixS1.docx. [file SJI-102-e70069-s001.docx]

**Oral and skin sensitisation to peanut show different immunological features in Brown Norway rats**

Tiffany K. S. Sztuk^1,a^, Jeppe M. Larsen^1,b^, Neil M. Rigby^2^, Anne-Sofie R. Ballegaard^1^, Irina Pozdnyakova^3^, Stef J. Koppelman^4^, Alan R. Mackie^2^, Katrine L. Bøgh^1^

^1^National Food Institute, Technical University of Denmark, Kgs. Lyngby, Denmark

^2^School of Food Science & Nutrition, University of Leeds, Leeds, UK

^3^NNF Center for Protein Research, University of Copenhagen, Copenhagen, Denmark

^4^Institute of Agriculture and Natural Resources, Food Science and Technology, University of Nebraska-Lincoln, Lincoln, USA

^a^Present address: BioAnalytics, ALK-Abelló A/S, Hørsholm, Denmark

^b^Present address: Department of Technology, University College Copenhagen, Copenhagen, Denmark.

**Supplementary information**

**1. Materials and methods**

**1.1 ELISA – quantification of antibody titres**

IgG1 and IgE specific for PPE, denatured (d.)PPE, Ara h 1, d.Ara h 1, Ara h 2, d.Ara h 2, Ara h 3, d.Ara h 3, Ara h 6 and d.Ara h 6 were quantified by means of indirect and antibody-capture ELISAs, respectively.

For IgG1 ELISAs, 100 µL of 0.5 µg/mL PPE, d.PPE, d.Ara h 1, Ara h 2, d. Ara h 2, Ara h 3, d. Ara h 3, Ara h 6 or d.Ara h 6 or 100 µL of 1 µg/mL of Ara h 1 in carbonate buffer (15 mM Na_2_CO_3_, 35 mM NaHCO_3_, pH 9.6) was added to 96-well plates (Maxisorp, Nunc, Roskilde, Denmark) and incubated overnight at 4°C. Plates were washed five times in PBS with 0.01% (w/v) Tween 20 (P1379, Sigma Aldrich, Darmstadt, Germany) (PBS-T) after each incubation step. Subsequently, 50 µL/well of two-fold serial dilutions of serum and positive and negative control samples diluted in PBS-T were added, and plates were incubated for 1 h at room temperature (RT). For detection, 50 µL/well of HRP-labelled mouse-anti-rat-IgG1 (3060-05, Southern Biotech, Birmingham, AL, US) diluted 1:20,000 (v/v) in PBS-T was added, and plates were incubated for 1 h at RT. To visualise the enzymatic reaction, 100 µL/well of 3,3’,5,5-tetramethylbenzidine (TMB)-one substrate was added, and plates were incubated for 12 min at RT. The reaction was stopped by adding 100 µL/well of 0.2 M H_2_SO_4_, and absorbance was measured immediately after at 450 nm with a reference wavelength of 630 nm using a microtitre reader (Gen5, BioTek Instruments, Winooski, VT, US). Antibody levels were expressed as log_2_ titres and defined as the interpolated dilution of the given serum sample leading to the mean absorbance for the negative control serum + 3 SD.

For IgE ELISAs, 100 µL/well of 0.5 µg/mL mouse anti-rat (HDMAB-123 HybriDomus, Cytotek, Hellebæk, Denmark) in carbonate buffer was added to 96-well plates (microtitre, Maxisorp, Nunc) and incubated overnight at 4°C. Plates were washed five times with PBS-T between each incubation step. To block unspecific binding, 200 µL/well of 3% (v/v) rabbit sera (S2500-500, Biowest, Nuaillé, France) in PBS-T was added, and plates were incubated for 1 h at 37°C. Subsequently, 50 µL/well of two-fold serial dilutions of serum and positive and negative control samples diluted in PBS-T were added, and plates were incubated for 1 h at RT. Next, 50 µL/well of 0.2 µg/mL of digoxigenin (DIG)-coupled PPE (10:1) or DIG-d.PPE (10:1), 0.2 µg/mL DIG-Ara h 1 (10:1) or DIG-d.Ara h 1 (10:1), 0.2 µg/mL DIG-Ara h 2 (10:1) or DIG-d.Ara h 2 (10:1), 0.2 µg/mL DIG-Ara h 3 (10:1) or DIG-d.Ara h 3 (10:1), or 0.1 µg/mL DIG-Ara h 6 (10:1) or DIG-d.Ara h 6 (10:1) in 3% (v/v) rabbit sera (Biowest) in PBS-T was added to the plates and incubated for 1 h at RT. For detection of DIG-coupled PPE or individual allergens bound to IgE, 50 µL/well of HRP-labelled sheep-anti-digoxigenin (Anti-Digoxigenin-POD 1633716001, Roche Diagnostics GmbH, Mannheim, Germany) diluted 1:1,000 (v/v) in PBS-T was added and plates were incubated for 1 h at RT. Visualisation was performed as described for IgG1 ELISAs. Antibody levels were expressed as log_2_ titres and defined as the interpolated dilution of the given serum sample leading to the mean absorbance for the negative control serum + 3 SD.

**2.2 Flow cytometry**

Single-cell suspensions were prepared from mesenteric lymph nodes (mLN), Peyer’s patches (PP), small intestine without PP (SI), abdominal skin draining lymph nodes (dLN), and abdominal skin for the analysis by flow cytometry, as previously described (1). SI tissue was collected as the 9 cm of SI following the first 29 cm of intestine distal from the stomach. PPs were removed and combined with PPs collected from the remaining part of the SI distal of the SI sample. The inguinal lymph nodes were collected as the lymph nodes (dLN) draining the abdominal skin. Abdominal skin (1 x 1 cm) was excised at the site of skin sensitisation. Additionally, whole blood samples were collected in heparin-coated tubes (BD Biosciences, Franklin Lakes, NJ, US).

SI samples were cut into 5 mm pieces using a scissor. The pieces were washed five times in wash buffer (PBS with 2% (v/v) heat-inactivated fetal calf serum (FCS, Sigma), and 15 mM HEPES). Skin tissue was cut into 2 mm pieces using a razor blade. SI and skin tissues were digested in RPMI medium (Sigma) with 10% (v/v) FCS, 15 mM HEPES (Sigma), 100 units/mL penicillin (Sigma), 100 μg/mL streptomycin (Sigma), 250 μg/mL collagenase (Sigma), and 1 mg/mL dispase II (Sigma) for 45 min on a horizontal tube rotator at 37°C. The digested tissues were lightly minced through a 70 μm cell strainer (BD Biosciences), which was regularly rinsed with FACS buffer (PBS with 2% (v/v) FCS and 0.05% (v/v) NaN_3_). mLN, PP, and dLN samples were lightly minced through a 70 μm cell strainer, which was regularly rinsed with FACS buffer. The cell suspensions were centrifuged at 400 *g* (mLN, SI, dLN, and skin samples) or 600 *g* (PP samples), at 4°C for 10 min. Cells were resuspended in FACS buffer and counted using a NucleoCounter (Chemometec, Allerød, Denmark).

Three different staining panels were used to analyse antigen presenting cells (panel 1), regulatory T cells (panel 2), and T cell skin and intestine homing receptor expression (panel 3):

Panel 1 (antigen presenting cells):

- CD161/FITC (clone 10/78, BD Biosciences)
- HIS36/PE (clone HIS63, BD Biosciences)
- CD103/AF647 (clone OX-62, BD Biosciences)
- CD3/PerCp-eF710 (clone eBioG4.18, Thermo Fisher Scientific)
- CD11c/b/PE-Cy7 (clone OX-42, BD Biosciences)
- CD45/APC-eF780 (clone OX-1, Thermo Fisher Scientific)
- RP1/BV421 (clone RP-1, BD Biosciences)
- RT1b/BV510 (clone OX-6, BD Biosciences)

Panel 2 (regulatory T cells):

- CD3/PerCp-eF710 (clone eBioG4.18, Thermo Fisher Scientific)
- CD4/PE-Cy7 (clone OX-35, BD Biosciences)
- CD45/APC-eF780 (clone OX-1, Thermo Fisher Scientific)
- CD25/BV421 (clone OX-39, BD Biosciences)
- CD8a/BV510 (clone OX-8, BD Biosciences)
- FoxP3/FTIC (clone JFK-16s, Thermo Fisher Scientific)
- T-bet/PE (clone O4-46, BD Biosciences)

Panel 3 (T cell homing receptors):

- CCR4/PE (clone 205410, R&D Systems)
- CCR9/APC (clone eBioCW-1.2, Thermo Fisher Scientific)
- CD3/PerCp-eF710 (clone eBioG4.18, Thermo Fisher Scientific)
- CD4/PE-Cy7 (clone OX-35, BD Biosciences)
- CD45/APC-eF780 (clone OX-1, Thermo Fisher Scientific)
- CD25/BV421 (clone OX-39, BD Biosciences)
- CD8a/BV510 (clone OX-8, BD Biosciences)

One million cells obtained from mLN, PP, SI, dLN, and skin samples were incubated with 50 μL blocking solution with 10% (v/v) rat serum and 5 μg/mL anti-CD32 (clone D34-485, BD Biosciences) in FACS buffer for 5 min on ice. Cells were subsequently surface stained by addition of 50 μL blocking solution containing a 1:100 dilution of the antibodies in flow panel 1, 2, or 3, and incubated 20 min on ice. In the preparation of flow panel 2, intercellular staining of FoxP3 and T-bet was performed using the BD Transcription Factor buffer set (BD Biosciences) according to the manufacture’s protocol with a 1:100 dilution of the antibodies.

Whole blood samples were diluted two times in FACS buffer, and 100 μL were stained with 50 μL FACS buffer containing a 1:100 dilution of the antibodies in flow panel 1, 2, or 3, and incubated for 20 min on ice. Red blood cells were subsequently lysed by adding 1 mL VersaLyse (Beckman Coulter, Brea, CA, US). The samples were incubated in the dark at RT for 10 min before 2 mL FACS buffer was added and samples were centrifuge at 350 *g*, at 4°C for 6 min. Cells were resuspended in FACS buffer. In the preparation of flow panel 2, intercellular staining of FoxP3 and T-bet was performed as described above.

Flow data was acquired using a BD FACSCanto II or BD LSRFortessa system (BD Biosciences) and analysed using FlowJo (version 10.7.1, BD Biosciences).

**2.3 Intestinal and skin gene expression**

To investigate mRNA expression levels in the SI, a 1 cm piece, 27 cm distal from the stomach, was excised from each group. To investigate mRNA expression levels in the skin, a 1 x 1 cm piece from the abdomen was excised. SI tissues and skin samples were stored in RNAlater (Invitrogen, Carlsbad, CA, US) overnight at 4^o^C and subsequently stored at -80^o^C until extraction of RNA. RNA extraction, followed by cDNA synthesis, was performed as previously described (1) with the exception of using the RNA lipid kit (74804, Qiagen, Hilden, Germany) for skin samples. Real time (RT)-quantitative polymerase chain reactions (qPCRs) were run with diluted cDNA (1:10). TaqMan gene assays used were *IL-10* (Interleukin 10 Rn99999012_m1), *TGF-β* (Transforming growth factor β Rn00572010_m1), *IFN-γ* (Interferron γ Rn00594078_m1), *IL-4* (Interleukin 4 Rn01456866_m1), *IL-5* (Interleukin 5 Rn01459975_m1), *IL-13* (Interleukin 13 Rn00587615_m1), *TSLP* (Thymic stromal lymphopoietin Rn01761072_m1), *IL-25* (Interleukin 25 Rn04244818_m1), *IL-33* (Interleukin 33 Rn01759835_m1), and *Ocln* (Occludin Rn00580064_m1). The relative gene expression was calculated using the 2^-ΔCT^ method with *B2m* (Beta-2-microglobulin Rn00560865_m1) (SI and skin samples) and *Sdha* (succinate dehydrogenase complex Rn00590475_m1) (SI samples) as normalisation genes. Data was acquired with Quantstudio 7 Flex software (Applied Biosystems, Foster City, CA, US).

**2. Results**

**
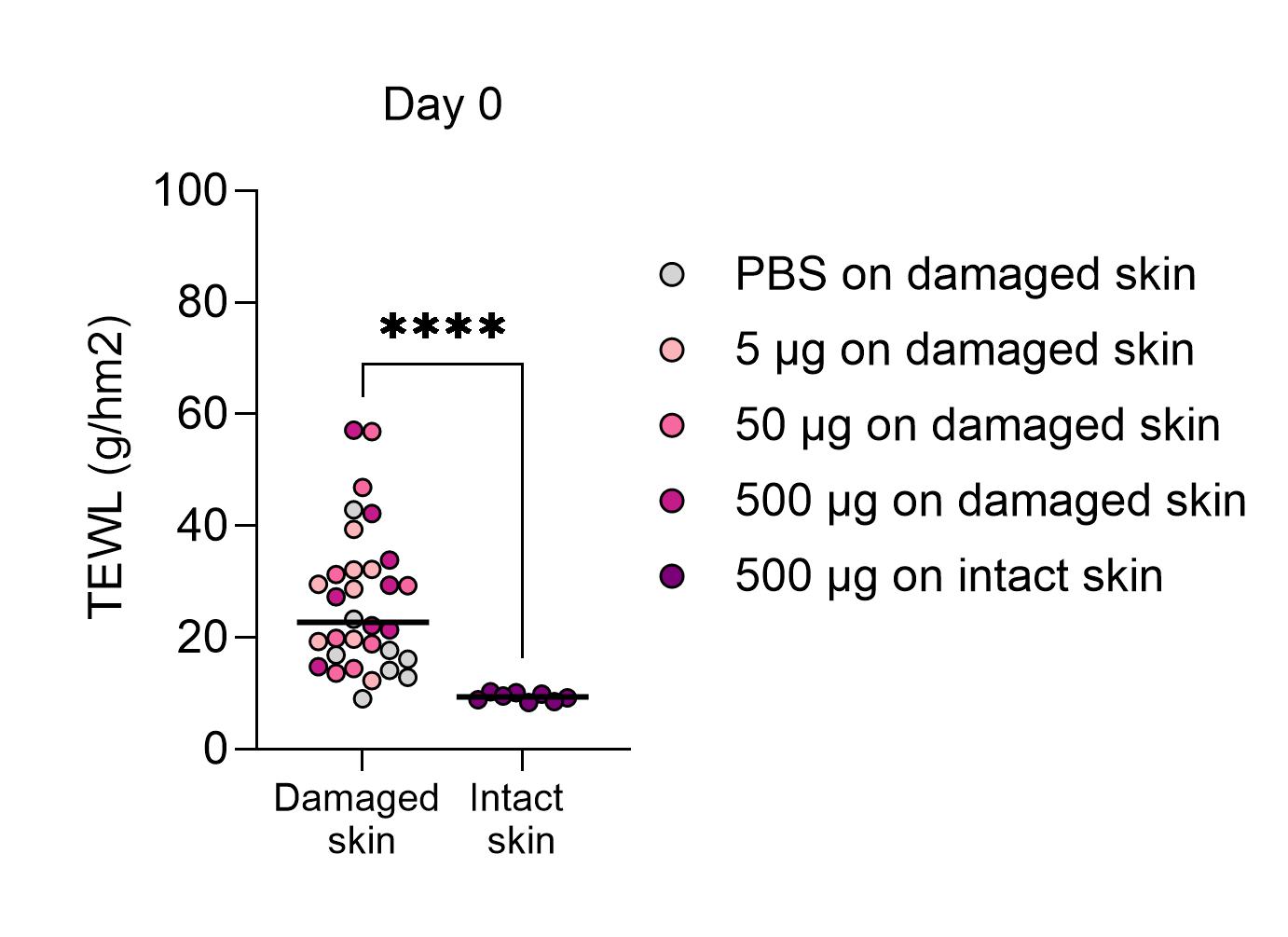
**

***FIGURE S1: Transepidermal water loss (TEWL) as a measure of the skin barrier integrity on Day 0.*** *The abdominal skin of each animal was either damaged by mechanical disruption or left intact prior to skin application with PBS, 5 µg, 50 µg or 500 µg of peanut protein extract. Each symbol represents a single animal, and horizontal lines indicate the median. The statistically significant difference in TEWL between animals with damaged and intact skin is indicated with asterisks, **** p<0.0001.*

**
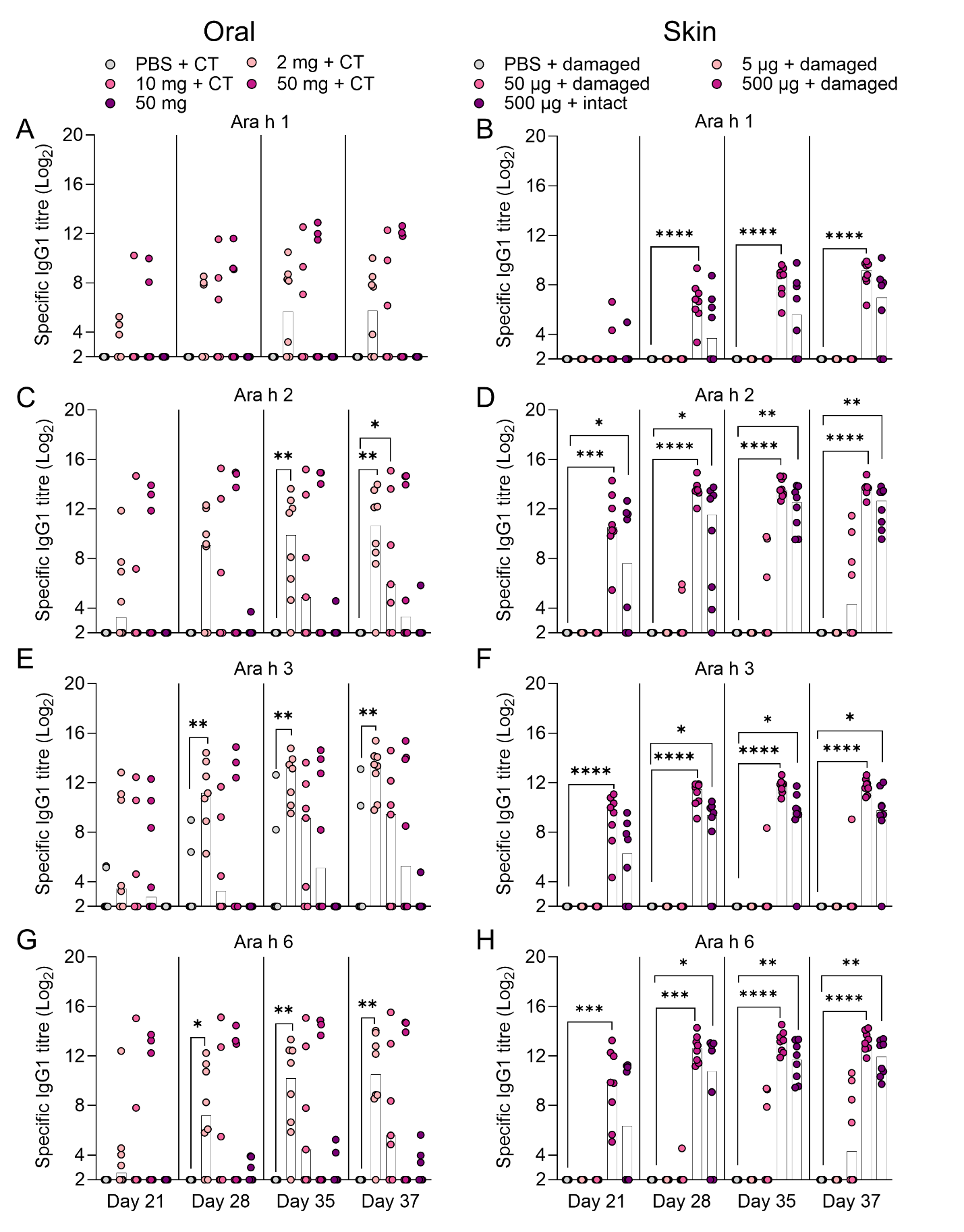
**

***FIGURE S2. IgG1 inducing capacity of peanut protein extract and the major peanut allergens.*** *Animals were sensitised orally by gavage with peanut protein extract (PPE) together with 20 µg cholera toxin (CT) or by skin administration of PPE in PBS on damaged or intact skin three times per week for five weeks. Animals receiving PBS as control were included for both administration routes. IgG1 titres for Ara h 1 (A+B), Ara h 2 (C+D), Ara h 3 (E+F) and Ara h 6 (G+H) in animal sera on Day 21, 28, 35 and 37 were determined by ELISA. Left panel shows the development of specific IgG1 responses for animals exposed to PPE via the oral route, and right panel shows the development of specific IgG1 responses for animals exposed to PPE via the skin route.* *Each symbol represents a single animal, and horizontal lines indicate the median of each group. Statistically significant differences compared to control animals receiving PBS are indicated with asterisk(s), * p<0.05, ** p<0.01, *** p<0.001, **** p<0.0001.*

**
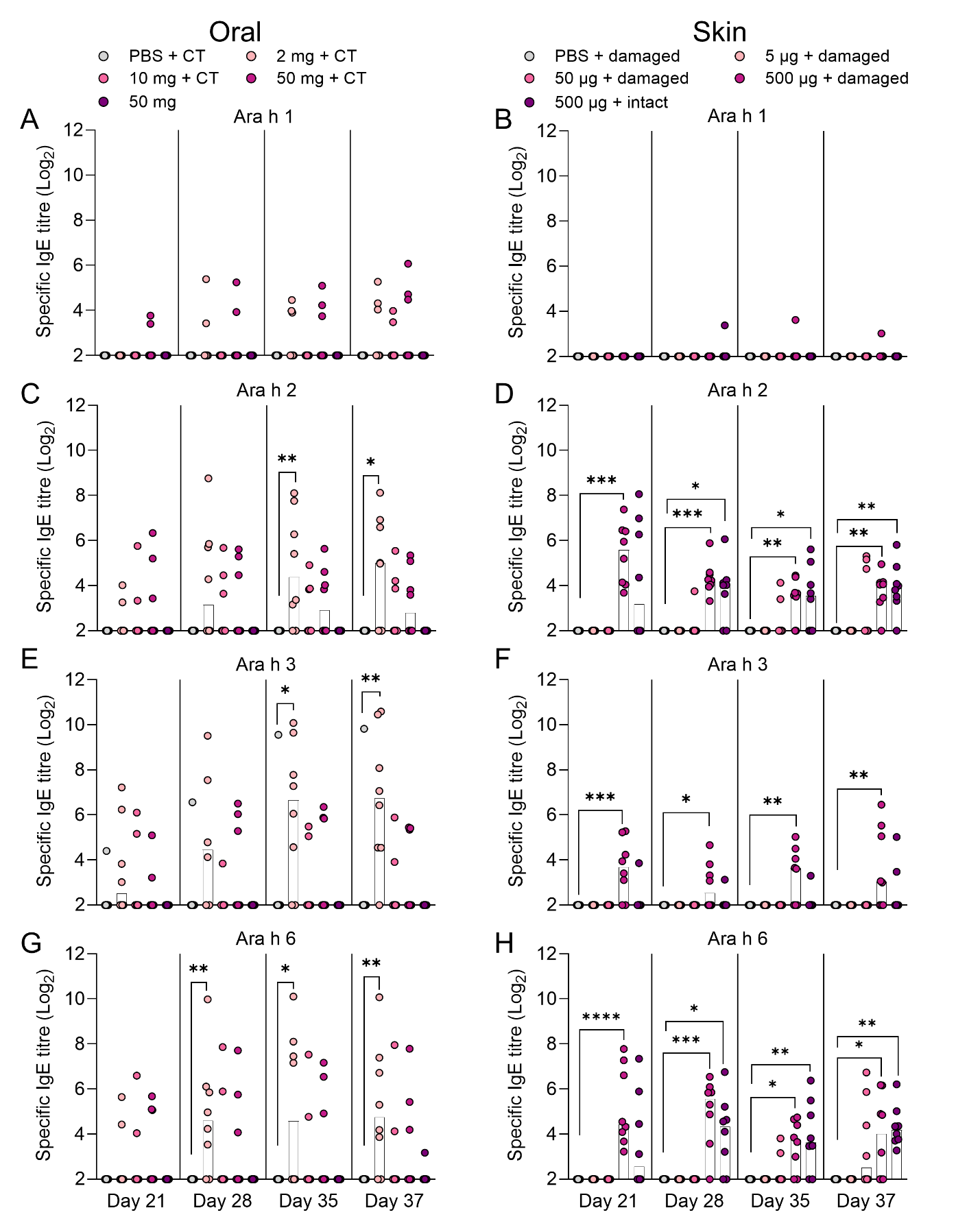
**

***FIGURE S3: Sensitising capacity of the major peanut allergens.*** *Animals were sensitised orally by gavage with peanut protein extract (PPE) together with 20 µg cholera toxin (CT) or by skin administration of PPE in PBS on damaged or intact skin three times per week for five weeks. Animals receiving PBS as control were included for both administration routes. IgE titres for Ara h 1 (A+B), Ara h 2 (C+D), Ara h 3 (E+F) and Ara h 6 (G+H) in animal sera on Day 21, 28, 35 and 37 were determined by ELISA. Left panel shows the development of specific IgE responses for animals exposed to PPE via the oral route, and right panel shows the development of specific IgE responses for animals exposed to PPE via the skin route. Each symbol represents a single animal, and horizontal lines indicate the median of each group. Statistically significant differences compared to control animals receiving PBS are indicated with asterisk(s), * p<0.05, ** p<0.01, *** p<0.001, **** p<0.0001.*

**
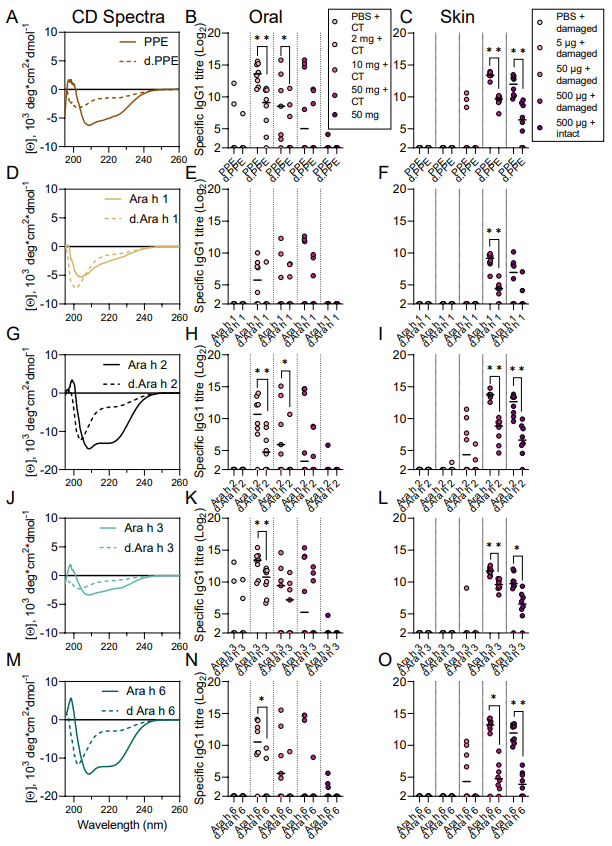
**

***Figure S4: Circular dichroism (CD) spectra and assessment of linear versus conformational IgG1 epitope recognition pattern.*** *Animals were sensitised orally by gavage with peanut protein extract (PPE) together with 20 µg cholera toxin (CT) or by skin administration of PPE in PBS on damaged or intact skin three times per week for five weeks. Animals receiving PBS as control were included for both administration routes. The levels of IgG1 specific for native or denatured (d.)PPE, Ara h 1, Ara h 2, Ara h 3 and Ara h 6 were assessed by ELISA on Day 37 in orally or skin sensitised animals as well as in control animals. Each symbol represents a single animal, and horizontal lines indicate median of each group. Statistically significant differences between recognition of conformational versus linear epitopes in orally or skin sensitised animals are indicated with asterisk(s), * p<0.05, ** p<0.01.*

**
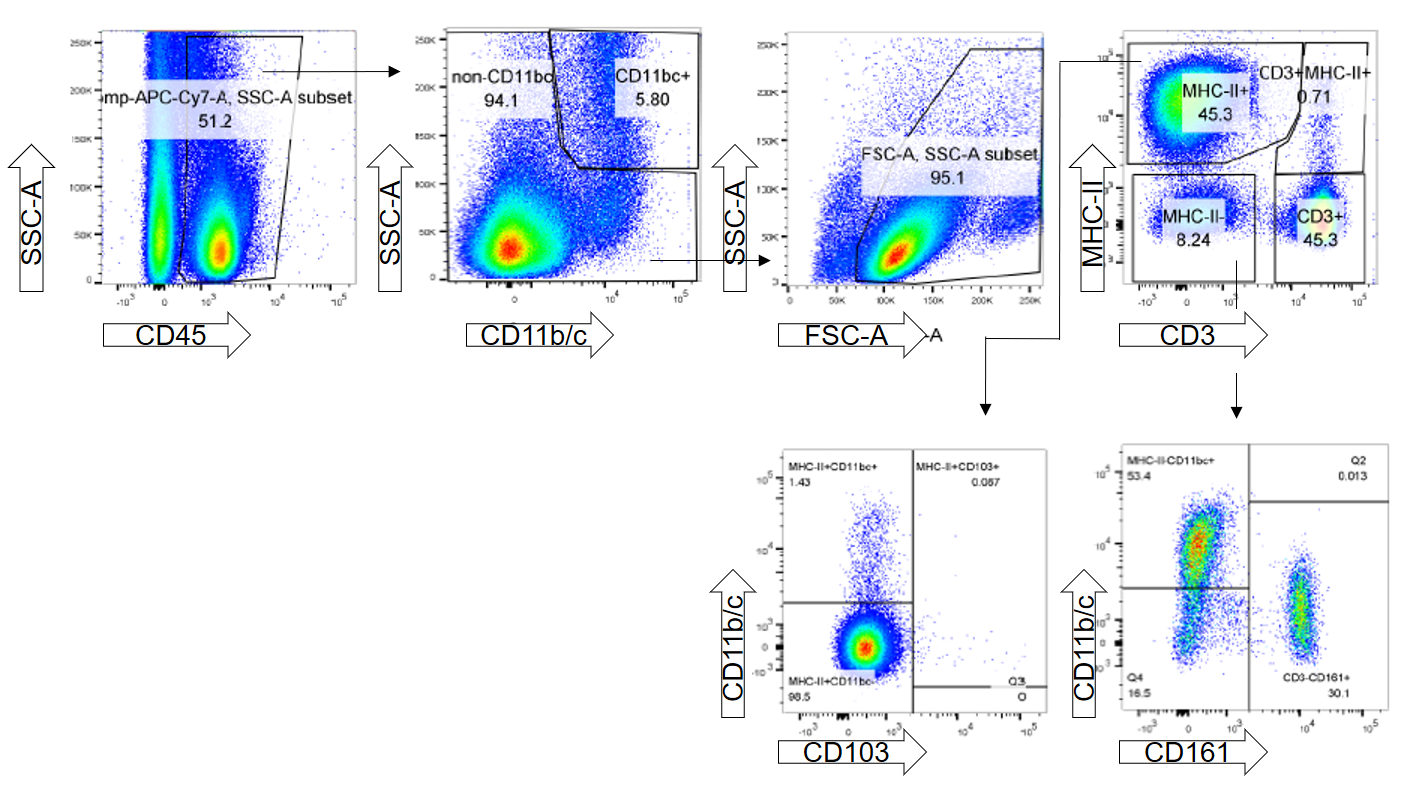
**

***Figure S5: Gating strategy for antigen presenting cells.*** *Populations of antigen presenting cells were determined by the CD45 and CD11b/c markers as well as by side and forward scatter area (SSC-A, FSC-A). This population was further divided into four subpopulations by the markers MHC-II and CD3. The non-T cell populations were further divided into populations identified by the markers CD11b/c, CD103 and CD161.*

*
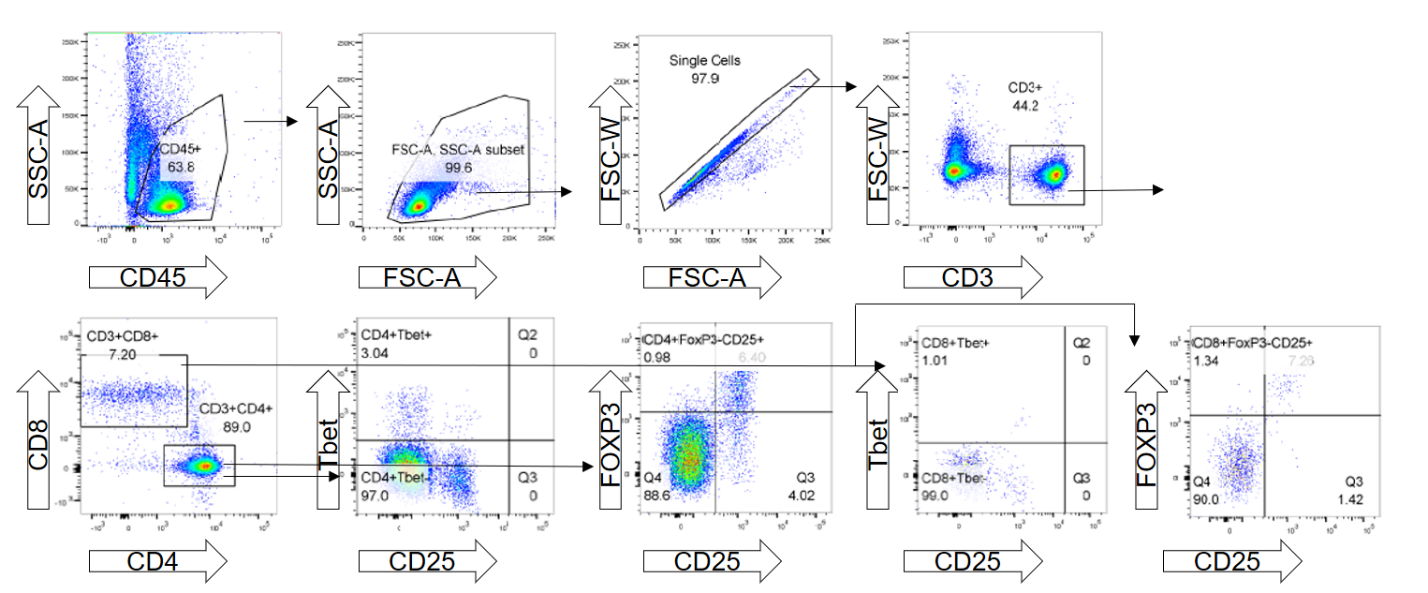
*

***Figure S6: Gating strategy for the regulatory T cell panel.*** *Single cell populations of the hematopoietic lineage were identified by the marker CD45 as well as by side and forward scatter area (SSC-A, FSC-A) and forward scatter width (FSC-W). T cells were identified by the CD3 marker and further subdivided into T helper cells and cytotoxic T cells by identifying the markers CD4 and CD8, respectively. Activated T cells were identified by the marker CD25, whereas T helper 1 cells and T regulatory cells were identified by the markers T-bet and FOXP3, respectively.*

**
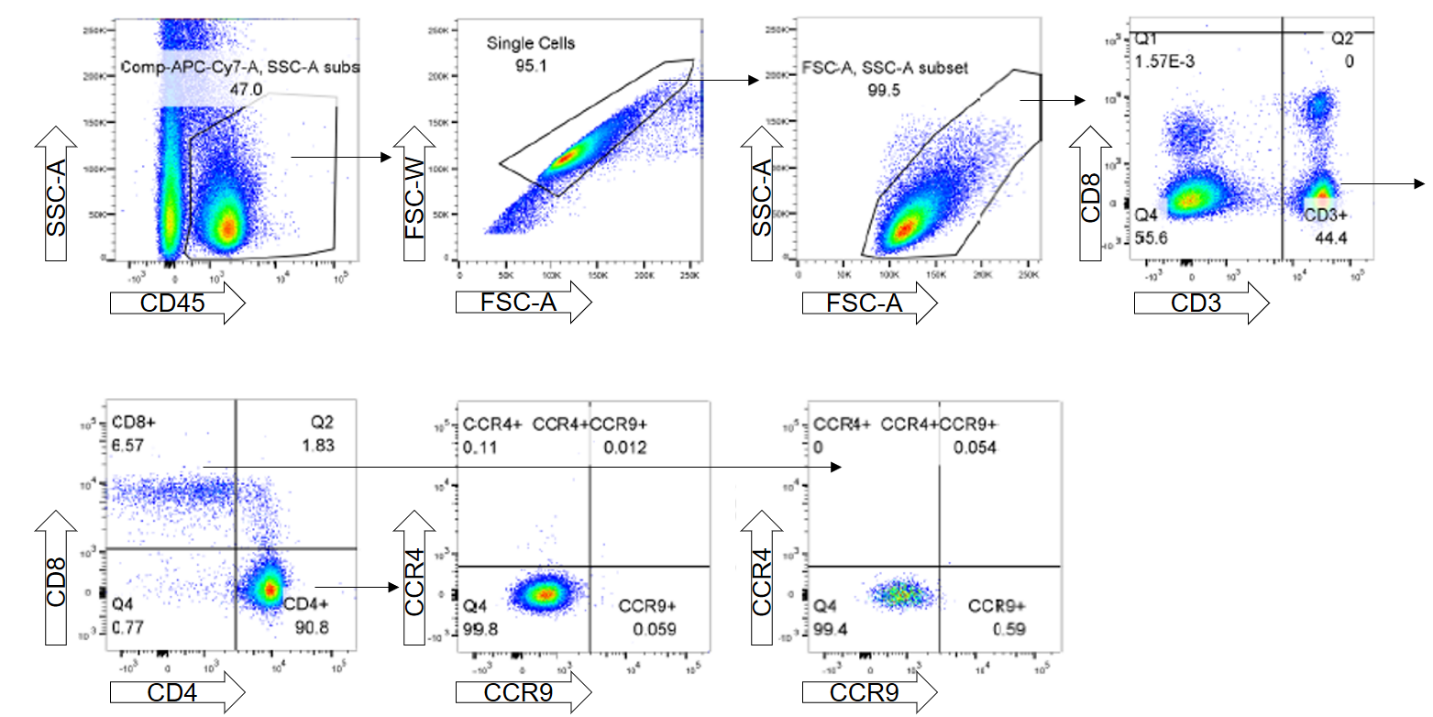
**

***Figure S7: Gating strategy for the homing panel.*** *Populations of single cells of the hematopoietic lineage were identified by the marker CD45, as well as by side and forward scatter area (SSC-A, FSC-A) and forward scatter width (FSC-W). T cells were identified by the marker CD3 and subsequently subdivided into T helper and cytotoxic T cells by the markers CD4 and CD8, respectively. Homing markers present on T helper and cytotoxic T cells were identified by the markers CCR4 and CCR9.*

***
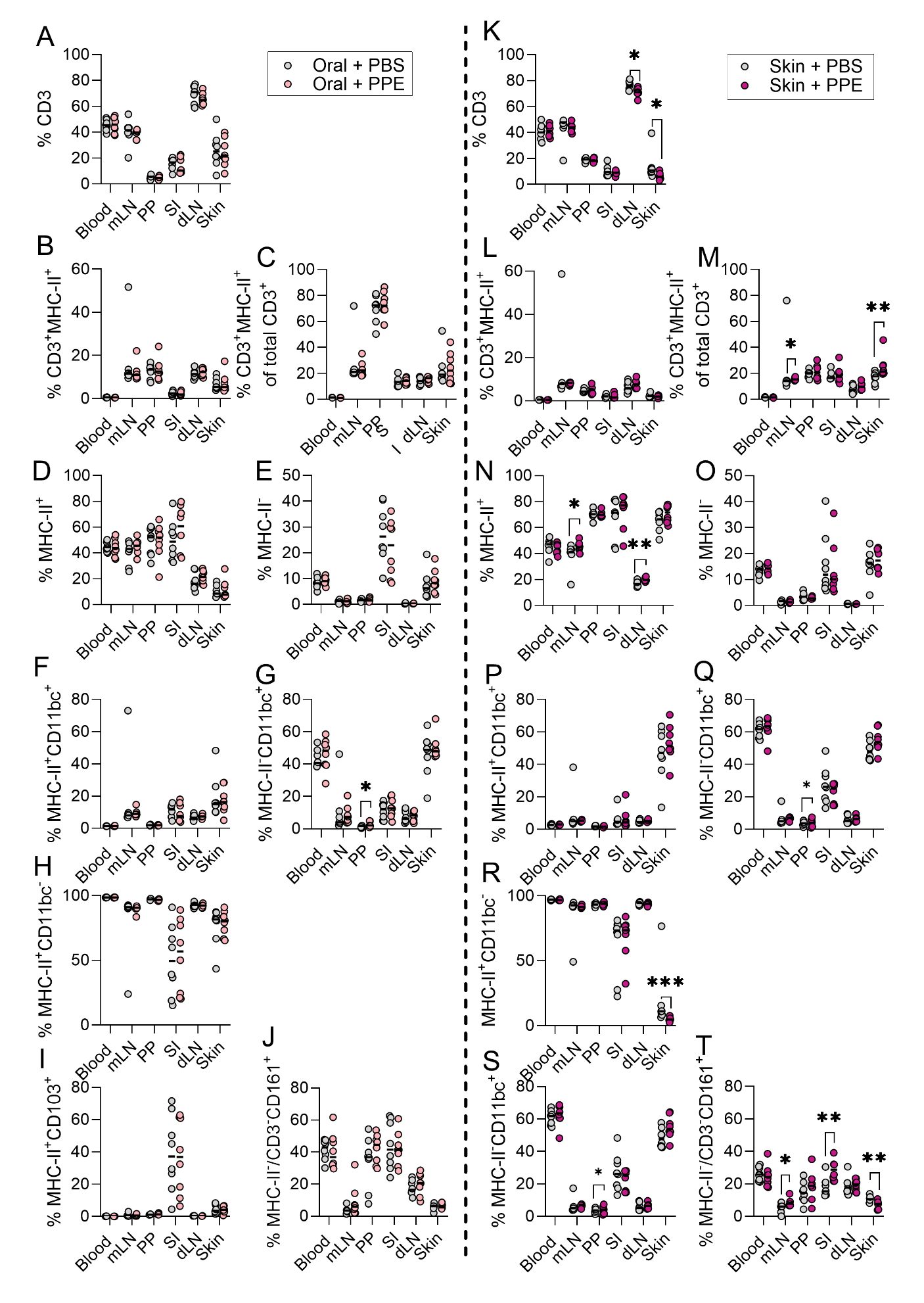
Figure S8: Flow cytometric analyses for the identification of antigen presenting cell populations induced in response to oral and skin sensitisation.*** *Animals were sensitised orally by gavage with 2 mg peanut protein extract (PPE) together with 20 µg cholera toxin (A-J) or by skin administration of 500 µg PPE in PBS on damaged skin (K-T) three times per week for five weeks. Animals receiving PBS as control were included for both administration routes. The different compartments included: blood, mesenteric lymph nodes (mLN), Peyer’s patches (PP), small intestine (SI), skin draining lymph nodes (dLN), and skin. Each symbol represents a single animal, and horizontal lines indicate median of each group. Statistically significant differences compared to control animals receiving PBS are indicated with asterisk(s), * p<0.05, ** p<0.01, *** p<0.001.*

***
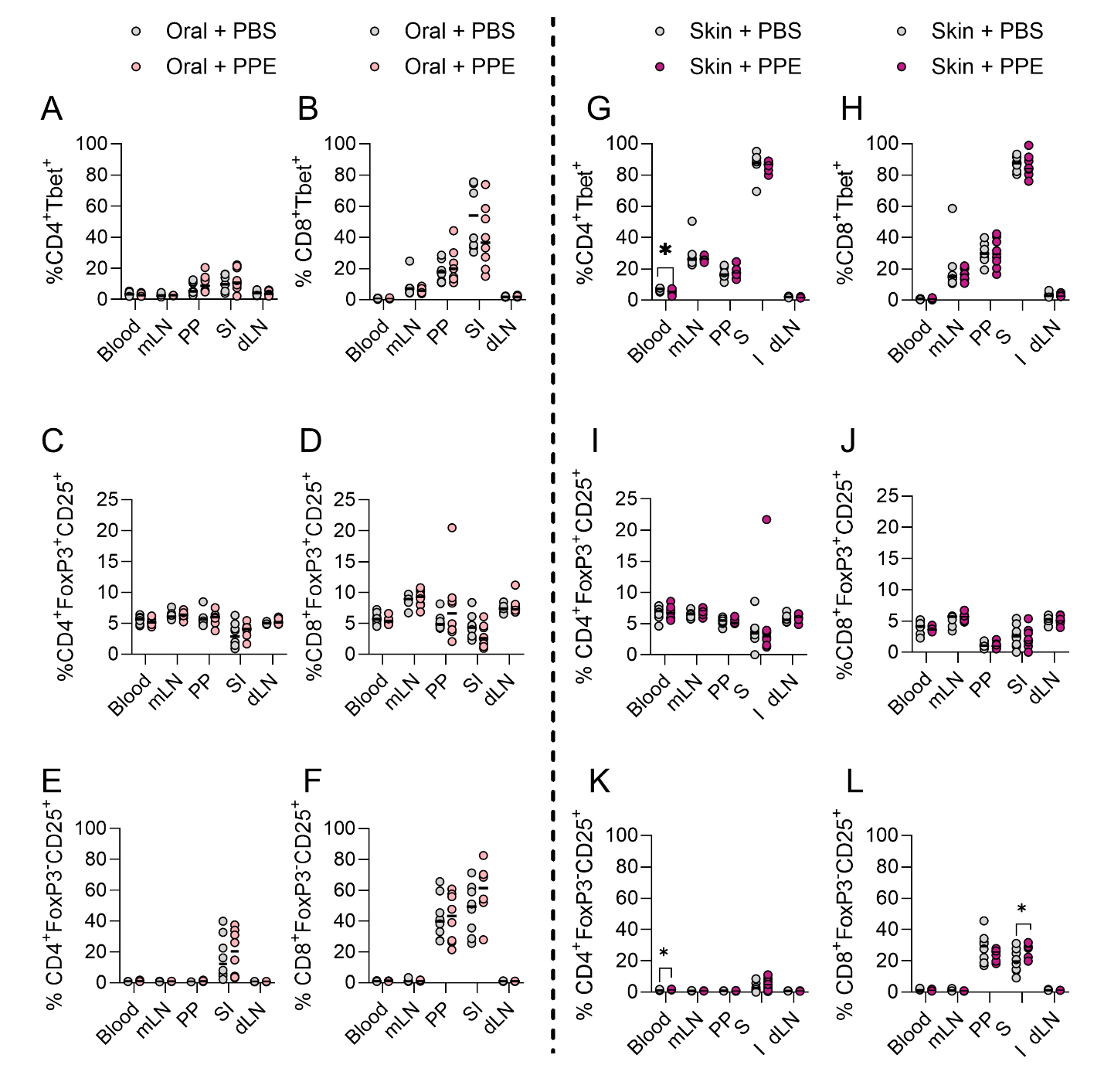
Figure S9: Flow cytometric analyses for the identification of cell populations with T regulatory phenotypic profiles induced in response to oral and skin sensitisation.*** *Animals were sensitised orally by gavage with 2 mg peanut protein extract (PPE) together with 20 µg cholera toxin (A-F) or by skin administration with 500 µg PPE in PBS on damaged skin (G-L) three times per week for five weeks. Animals receiving PBS as control were included for both administration routes. The different compartments included: blood, mesenteric lymph nodes (mLN), Peyer’s patches (PP), small intestine (SI), skin draining lymph nodes (dLN), and skin. Each symbol represents a single animal, and horizontal lines indicate median of each group. Statistically significant differences compared to control animals receiving PBS are indicated with an asterisk, * p<0.05.*

**
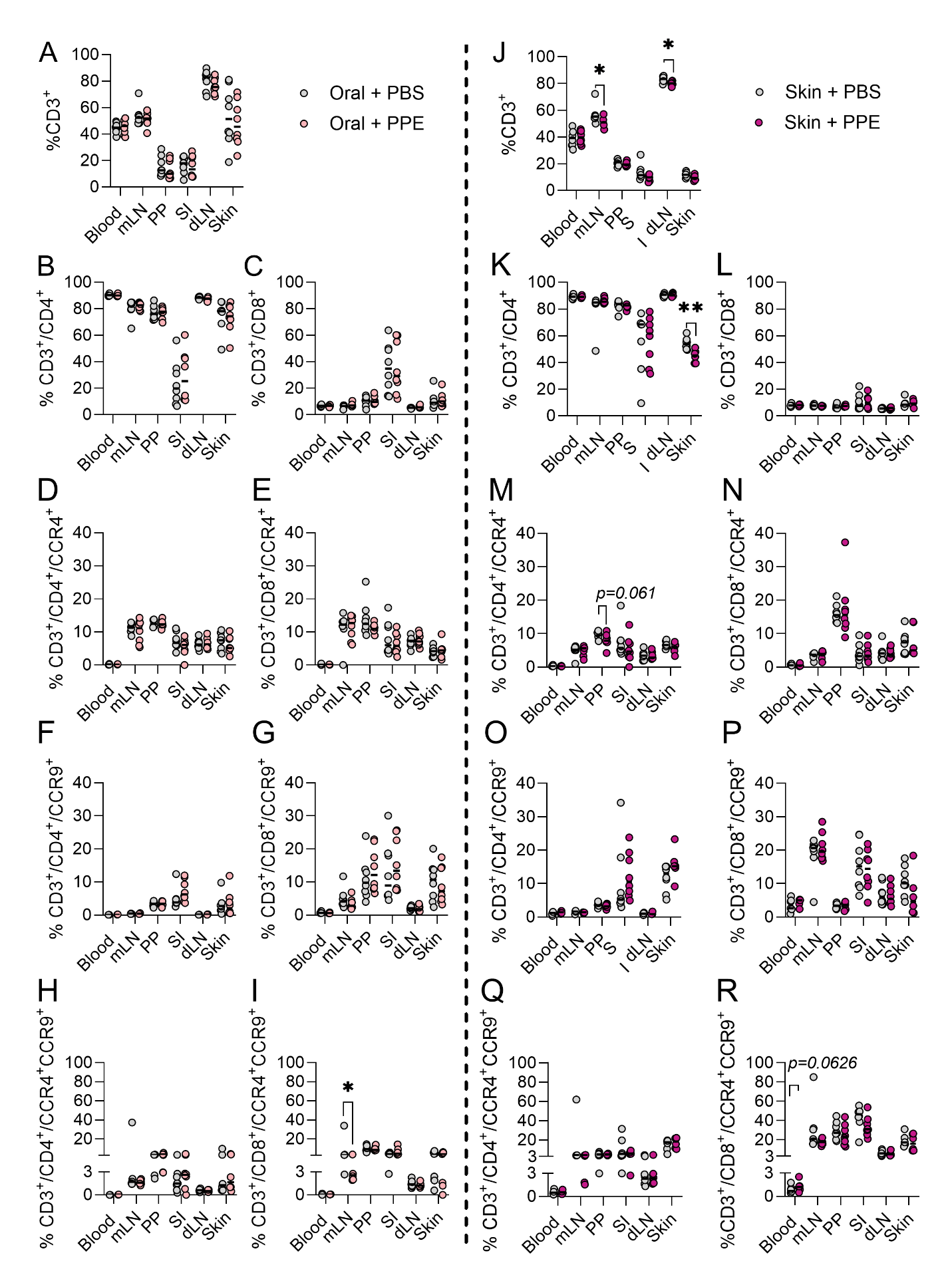
**

***Figure S10: Flow cytometric analyses for the identification of cell populations with homing properties induced in response to oral and skin sensitisation.*** *Animals were sensitised orally be gavage with 2 mg peanut protein extract (PPE) together with 20 µg cholera toxin (A-I) or by skin administration of 500 µg PPE in PBS on damaged skin (J-R) three times per week for five weeks. Animals receiving PBS as control were included for both administration routes. The different compartments included: blood, mesenteric lymph nodes (mLN), Peyer’s patches (PP), small intestine (SI), skin draining lymph nodes (dLN), and skin. Each symbol represents a single animal, and horizontal lines indicate median of each group. Statistically significant differences compared to control animals receiving PBS are indicated with asterisk(s), * p<0.05, ** p<0.01.*

**
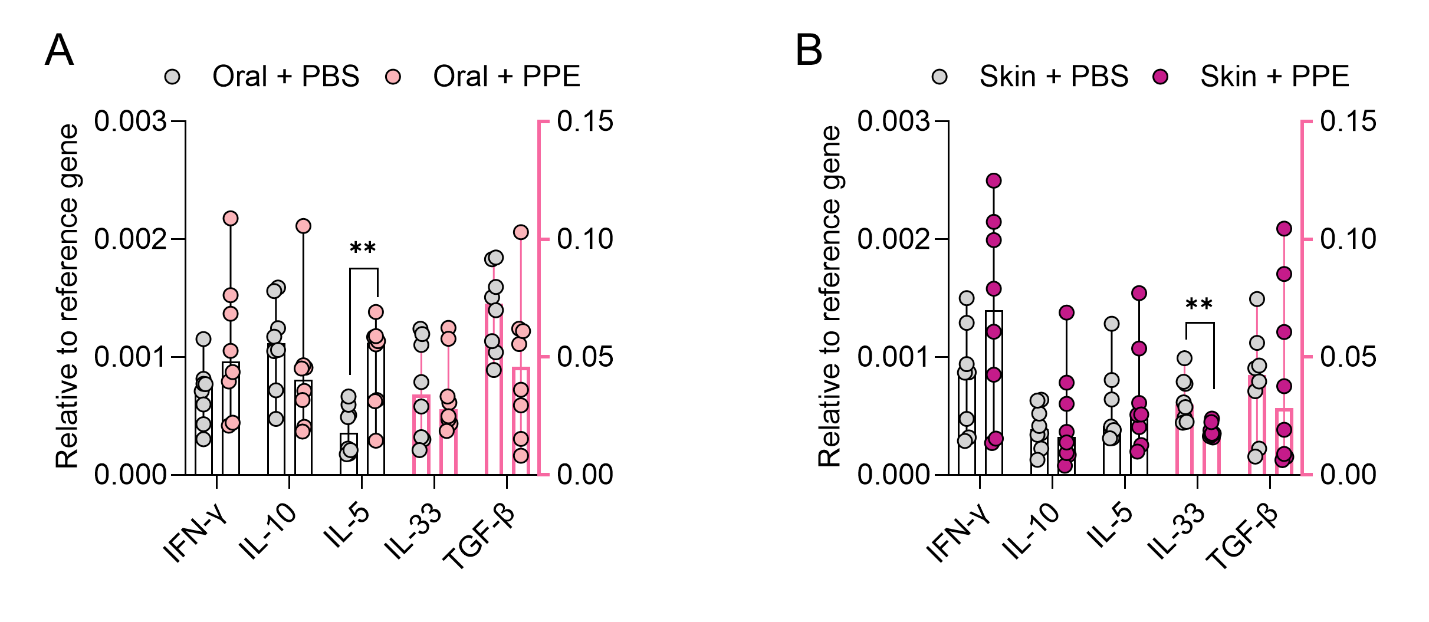
**

***Figure S11. Relative gene expression of different markers in skin samples from orally and skin sensitised animals.*** *Animals were sensitised orally by gavage with 2 mg peanut protein extract (PPE) together with 20 µg cholera toxin (A) or by skin administration of 500 µg PPE in PBS on damaged skin (B) for three times per week for five weeks. Animals receiving PBS as control were included for both administration routes. Gene expression of interleukins (IL), interferon (IFN)-γ and transforming growth factor (TGF)-β were determined by real-time quantitative polymerase chain reaction (RT-qPCR) and expressed relatively to B2m as the reference gene. Each symbol represents a single animal, and bars indicate median of each group. Statistically significant differences compared to control animals receiving PBS are indicated with asterisks, ** p<0.01.*

**3. References**

1. Graversen KB, Bahl MI, Larsen JM, Ballegaard A-SR, Licht TR, Bøgh KL. Short-Term Amoxicillin-Induced Perturbation of the Gut Microbiota Promotes Acute Intestinal Immune Regulation in Brown Norway Rats. *Front Microbiol* 2020;**11**:1–14.
